# The Early Diagnosis of Small-Pox

**Published:** 1896-08-22

**Authors:** J. A. Philip


### Medical Progress and Hospital Clinics.

[*The Editor will be glad to receive offers of co-operation and contributions from members of the profession. All letters should be addressed to* The Editor, at the Office, 428, Strand, London, W.C.]

### THE EARLY DIAGNOSIS OF SMALL-POX.

By J. A. Philip, M.A., M.D.

The early diagnosis of small-pox is not so easy as it is desirable that it should be, considering its importance. It has in common with certain other diseases, for which it may be, and has been often, mistaken, symptoms which precede and accompany the appearance of the eruption, and the eruption has at first a certain resemblance to some other eruptions. It is my object to draw attention to the points which it is important to bear in mind when we have to form a diagnosis in cases which may, or may not, prove to be small-pox. Of course it is impossible in many cases to pronounce with accuracy the nature of such a disease before the appearance of the eruption, but it is often desirable to form an opinion as to the probable nature of the illness. In doing so we have naturally to consider the question of infection, and it should be borne in mind that though the two diseases with which small-pox is liable to be confounded at the first appearance of the rash—viz., chicken-pox and measles—are frequent visitors, small-pox, on the other hand, is an infrequent one, and unless there is something in the surroundings of the patient to render not improbable an infection by persons or objects coming from a distance, it is most unlikely that, during the absence of small-pox in the locality, we have a case of this to deal with.

As a consequence of the infrequency which I have mentioned medical men may be in practice for many years without seeing a case of small-pox, and they lack the confidence which acquaintance with the disease can alone give. The constitutional disturbance which we see during the pre-eruption period does not differ materially from that seen in many diseases, such as influenza, typhus fever, &c., but I propose to confine myself to a comparison between small-pox, chicken-pox, and measles. In each of these we may have the usual symptoms that characterise the onset of a fever, such as malaire, aching of the limbs, loss of appetite, shivering, rise of temperature, and accelerated pulse. Such symptoms are common to all, and in each they may vary in degree, being sometimes so slight as to pass unnoticed. We shall see now how far these vary in severity and in type in the three diseases under consideration.

Taking small-pox first, we find that these symptoms are, as a rule, more marked than in the others. They consist of: (1) Severe rigors or a succession of chills, often marking the onset. (2) Violent pains, like those of rheumatism, most frequently felt in the back, head, or thighs. (3) Vomiting is a marked and frequent symptom. It may be only occasional or very persistent. (4) Prostration is not infrequent, and may be very marked, with cold skin and sunken features. (5) The skin may be dry and burning, or bathed at times in perspiration. This is a useful point to bear in mind. (6) The pulse may be rapid and the breathing hurried. (7) There may be inflammation of the oculo-nasal mucosa, with lachrymation, sneezing, &c. (8) A very interesting and highly characteristic sign is the appearance of a pre-eruption rash. This is totally different from the small-pox rash, and varies greatly in site and character. Sometimes we have a dusky brownish stippling in the flexures of some of the joints; at others a brick-red punctated coloration of some surface of the body, preferably the triangular area below the level of the umbilicus. This often becomes the site of the petechiæ in hæmorrhagic small-pox. (9) In hæmorrhagic small-pox we may see before the papules appear small petechiæ like flea-bites, which rapidly increase in number, and may be attended by hæmorrhagic effusions under the conjunction or from the mucous surfaces. In such cases death may occur before the eruption has begun, as seen in the illustrations done by me in "Collie on Fevers."

How far do these symptoms correspond with, or differ from those of chicken-pox or measles? Rigors do not, as a rule, characterise the onset of the latter diseases, but they have been seen in cases of chicken-pox occurring in elderly persons. The severe pains are decidedly characteristic of small-pox, as are vomiting, prostration, and profuse sweating. The pre-eruptive rashes and the hæmorrhagic petechiæ are seen only in small-pox. I may mention that I once saw hæmorrhagic effusion into the vesicles of a case of mild chicken-pox.

The *ensemble* of symptoms is of a decidedly milder type in chicken-pox and in measles, and not infrequently the first thing noticed is the appearance of the rash. However, this is not always the case; and, especially in adults, the constitutional disturbance may be as marked as in most cases of small-pox.

It is a mistake to consider chicken-pox as confined to youth. I have notes of cases at all ages, and last year I saw a patient suffering from it at 50 years of age, where the deviation from the familiar character of the eruption, as seen in children, was so marked in parts, such as the face and hands, that it was only after careful examination of other parts of the body, and study of the history, that I felt justified in pronouncing it a case of chicken-pox, particularly as small-pox was prevalent at the time.

One symptom is common to measles and small-pox—namely, injection of the oculo-nasal mucosa. In the former it begins earlier, and is attended with profuse lachrymation, sneezing, and a loud characteristic cough. These may last for some days before any rash is seen. The secretion in small-pox is usually of a more sticky, yellowish character, and the eyes only are markedly affected. This affection of the mucosa begins much later in small-pox. If the throat is examined, bright red papules will be seen on the velum palati, and other parts of the throat, about twenty-four hours before the eruption on the skin.

We come now to the inset of the eruption in the three diseases.

In smallpox the aspect of the skin varies according to the abundance of the rash. In confluent and semi-confluent cases the small papules which appear give a swollen, heavy aspect and feel to the skin, which assumes the flush characteristic of habitual drunkards. When scanty, the skin is unaffected, except in the vicinity of the papules. These are seen first on the head and wrists, and rapidly spread downwards. Where the skin is thick, the papules become more prominent but less bright-red than in the protected regions of the body. They feel decidedly hard. The former group is most apt to be mistaken for measles, or *vice versâ*, the diffused blush with suffused eyes of the two being rather similar.

The measles rash appears first on the face and wrists, and is distinctly elevated, as in small-pox. At the junction of the face with the neck, and on the neck, the spots are of a brighter red and less raised, and the same applies to all the parts usually covered by clothes. The forehead becomes distinctly puffy during the first day, and on passing the finger over the cheeks the papules are distinctly felt, but are less hard than those of small-pox. The reddening of the papules soon increases and spreads into blotches, which run into each other. When the eruption is well out the measles rash can hardly be mistaken for small-pox, owing to the papules in the former having none of the induration noticed in the latter. We have also the noisy laryngeal cough in measles.

The chicken-pox eruption may appear particularly in children without any previous constitutional disturbance, and the first thing noticed is the appearance of a number of small red spots scattered over the head, trunk, and limbs. They are bright-red, and slightly elevated, but where the skin is thick they are larger and harder. Within a few hours fluid is secreted in many of them, and raises a thin layer of cuticle in a small hemispherical bulk, which bursts readily from pressure or rubbing, leaving a fine brown scab. Fresh crops keep coming out for four or five days, so that you may see papules, vesicles, and scabbed papules. This eruption is somewhat like that in mild cases of small-pox, but the points of difference are as follows: The smallpox eruption comes out nearly simultaneously, so that the whole of the eruption is nearly at the same stage. That of chicken-pox comes out in successive crops, so that you may see the three stages I have mentioned. In chicken-pox vesication begins within a few hours, and is quite unlike that of small-pox. The distribution of the two eruptions is very different. That of chicken-pox is distinctly more abundant on the trunk, especially on the back, whilst that of small-pox shows no such preference. In chicken-pox some of the vesicles on the trunk are larger than the majority of the crop, and are of an oval shape. The long axis of these large vesicles is always horizontal. Some of the vesicles in the head, on the face, or on the hands, owing to thickness of their covering, may never burst, and the fluid is slowly reabsorbed. These bear a very considerable resemblance to the vesicles of small-pox as they are of considerable size and very prominent.

It is not possible to diagnose small-pox before the appearance of the eruption, and one can always delay until the eruption has appeared. Then, with a careful study of the nature of the rash and of the history, &c., even one unfamiliar with the aspect of the diseases under review can always satisfy himself as to its nature. If he makes an error he may console himself by knowing that very many have made similar mistakes, that other diseases have been taken as small-pox, such as scarlet fever, shingles, syphilides, copaiba, rash, &c. Of course, some of these mistakes are not due to ignorance, but to great carelessness, and nothing in the way of instruction will prevent them; but sometimes medical men, even of high standing, have felt unable to pronounce a confident diagnosis after the appearance of the rash from lack of practical experience of the aspect of small-pox. As a matter of fact, I have found the class of medical men who make the fewest blunders in diagnosing small-pox are the parochial medical officers in a large town, whose practical knowledge keeps them right, whilst the theoretical instruction of holding posts in large metropolitan hospitals does not protect them from making mistakes.

Until a few years ago facilities were not provided for studying fevers, in spite of the vast importance of acquaintance with them; but the opening of the metropolitan fever hospitals to students will tend to remove this defect, and compulsory clinical instruction may save a young medical man on a future occasion from a *mauvais quatre d'heure* when he feels puzzled over a doubtful case.

